# Physician Perspectives on ChatGPT-4o as a Patient Resource for Abdominal Cancer Surgeries: Cross-Sectional Survey

**DOI:** 10.2196/81374

**Published:** 2026-05-04

**Authors:** Christina V Lindsay, Devika A Shenoy, Allison N Martin, Christie L Clipper, Kevin N Shah, Michael E Lidsky, Daniel P Nussbaum, Ralph Snyderman

**Affiliations:** 1 School of Medicine, Duke University, 8 Searle Center Dr, Durham, NC, 27710, United States, 1 305-439-7523; 2 Center for Personalized Health Care, Duke University, Durham, NC, United States; 3 Department of Surgery, Duke University, Durham, NC, United States; 4 Department of Medicine, Duke University, Durham, NC, United States

**Keywords:** patient education, health literacy, generative artificial intelligence, surgical oncology, perioperative care

## Abstract

**Background:**

Artificial intelligence (AI) models are being increasingly integrated into clinical care. Moreover, the availability of publicly accessible AI resources makes them attractive to patients seeking clinical information. Little is known regarding the use of large language models as patient resources for navigating major cancer diagnoses.

**Objective:**

This study aimed to evaluate the content, readability, and safety of ChatGPT (OpenAI; GPT-4o)-generated responses to common perioperative queries about hepatic, pancreatic, and colon cancers.

**Methods:**

A 28-question survey was developed based on frequently asked surgical questions for select malignancies. Surgical oncologists rated ChatGPT-4o-generated responses on a 5-point Likert scale for accuracy, quality, and tangibility. Readability was assessed using the Flesch-Kincaid Reading Grade Level (FKRGL) and Flesch Reading Ease (FRE). Respondents provided free-text comments and reported their comfort with patients using ChatGPT. Survey completion implied consent.

**Results:**

A total of 7 attending surgical oncologists with a median of 7 (IQR 4-13) years in practice completed the survey. Responses received mean scores of 3.5/5 (SD 0.28) for quality, 3.6/5 (SD 0.34) for accuracy, and 3.6/5 (SD 0.29) for tangibility. The responses had a median FKRGL score of 14.6 (IQR 13.3-15.6) and FRE score of 29.4 (IQR 20.5-36.3). On a post hoc analysis for select questions, the median FKRGL was 15.6 (IQR 14.4-16.7), decreasing to 7.1 (IQR 6.1-8.3) and 14.5 (IQR 13.2-15.4) with prompting and rephrasing, and the median FRE was 18.1 (IQR 14.6-24.7), increasing to 73.8 (IQR 66.6-79.3) and 32.0 (IQR 27.0-37.7) with prompting and rephrasing. Numerous inaccuracies and content gaps were reported, and approximately 43% (3/7) of providers did not report feeling “comfortable” in having patients consult publicly available AI for medical information.

**Conclusions:**

This study provides cautionary, yet optimistic, findings regarding the value of publicly accessible ChatGPT as a patient resource for abdominal malignancies. Providers should be prepared to effectively counsel patients to identify their educational attainment level when using ChatGPT to mitigate readability challenges.

## Introduction

In recent years, artificial intelligence (AI) has promised to reshape medicine. Chatbots such as ChatGPT (OpenAI) [1], DeepAI, and Google Gemini use large language models (LLMs), a popular form of AI. These models are trained upon large datasets to generate answers [2]. Recent LLM improvement in reasoning has been noted to reflect human-level cognition [3]. Furthermore, studies have examined LLM function in the health care sector. LLMs have been found to pass United States Medical Licensing Examinations [4,5] and medical subspecialty exams [6-8] and to provide successful clinical reasoning and diagnoses [9]. Moreover, ChatGPT has the potential to supersede other search engines in answering patient health–related questions by providing more comprehensive and specific answers [10,11].

Although AI has been found to augment medical practice, its use as a resource by patients is not well understood. Patients have long reported turning to the internet for clinical advice [12]. Studies evaluating responses from common search engines to frequently asked general surgery questions have typically found the quality to range from fair to good but found that the readability level often exceeded the recommended level for the general population [13]. More recently, patients have turned to ChatGPT for clinical questions; a study conducted in Australia found that approximately 9.9% of Australian adults asked ChatGPT medical questions within the first half of 2024 [14]. Following the rapid rise of publicly accessible proprietary LLM chatbots and the lack of peer-reviewed output within these learning models, recent work across specialties, including oncology, gastroenterology, otolaryngology, and surgery, has sought to evaluate LLM-generated responses to questions commonly asked by patients [15-21]. The reported overall quality of generated responses varies across fields and is impacted by the type of LLM used [20]. Furthermore, prior research has suggested that, as with “Dr Google” and other popular search engines [13], the readability of LLM-generated responses may serve as a key limitation of using LLMs such as ChatGPT as a patient resource [22]. Additionally, ChatGPT answers are limited in consistency [23], generating similar but nonidentical responses.

Gastrointestinal malignancies, including pancreatic, colorectal, hepatic, stomach, and esophageal malignancies, account for over one-quarter of cancer incidence globally and are steadily increasing. By 2040, the global number of gastrointestinal cancer deaths is projected to increase by over 70% to 5.6 million [24]. Given the significant disease burden of gastrointestinal malignancies and related therapies, it is essential to properly evaluate pertinent patient resources to better inform patients, many of whom will be accessing these resources independently. To date, few studies have examined the use of publicly accessible proprietary LLMs as a perioperative resource for patients with abdominal malignancies. The aim of this study was to evaluate the content and readability of LLM-generated responses to common patient queries for hepatic, pancreatic, and colon cancers.

## Methods

### Ethical Considerations

This study was submitted to the Duke University Institutional Review Board for review and was determined to be exempt (Pro00116649). This study involved surgeon-participants who evaluated GPT-generated responses to frequently asked questions. No patient data were used. To maintain participant confidentiality, all data were analyzed in aggregate. All GPT inquiries and survey questions were asked in English. Consent from participants was implied through voluntary completion of the survey. No compensation was provided to participants.

### Question Development

Preliminary questions were developed by CVL and DAS. Questions for this study were developed by sourcing frequently asked questions about colon, liver, and pancreatic cancers from 7 hospital patient information and nonprofit cancer foundation websites [25-31]. This methodology was used in an earlier study examining LLMs as a tool for patient education in lung cancer surgery [32]. Questions on general disease information, including signs and symptoms, staging and treatment options, surgical eligibility, and operative risks, were formulated for colon, hepatic, and pancreatic cancers using identical language for each condition. Standardized language was used to determine the suitability of LLMs for delivering useful abdominal cancer education as applied to each of the conditions. Additional questions were created to address common patient concerns related to postoperative recovery and potential adverse outcomes following abdominal cancer surgery.

### Question Piloting

The preliminary survey questions were initially evaluated for relevance and alignment with patient phrasing through subjective assessment by 2 general surgery residents. Residents were prompted to assess the frequency with which the proposed questions were encountered in practice to evaluate the survey questions based on clinical relevance. Residents were also prompted to evaluate the survey questions based on alignment with patient phrasing, to suggest phrasing revisions for items that received a Likert score ≤3 on a 5-point scale, and to propose additional relevant questions not addressed by the survey. At this stage, 8 questions were removed and 4 were added per resident feedback. Additional questions were adjusted accordingly. After piloting scenarios with residents, all questions were run sequentially through a publicly available, proprietary version of ChatGPT (GPT-4o; released on May 13, 2024) [1,33], on March 9, 2025, in Durham, United States. ChatGPT-4o is based on a proprietary GPT-4-class pretrained base LLM that has been instruction-tuned for conversational use. No additional model fine-tuning or retraining was performed through this study. ChatGPT was prompted to answer in paragraph form without additional contextual information. As in prior studies, a new chat entry was posed for each question [32].

A Qualtrics survey was formulated with the final 28 questions and LLM responses. This survey was piloted by our surgeon expert, ANM, who provided final revisions for question phrasing. Table 1 lists the finalized questions prompted into ChatGPT. Revised questions were newly run through ChatGPT, and the Qualtrics survey was adjusted accordingly.

**Table 1.** GPT queries: abdominal cancer frequently asked questions and common postoperative complications.

Domain	Question
Signs and symptoms	Q1: What are the signs and symptoms of pancreatic cancer? Q2: What are the signs and symptoms of colon cancer? Q3: What are the signs and symptoms of liver cancer?
Stages and treatment	Q4: What are the different stages and treatments for pancreatic cancer? Q5: What are the different stages and treatments for colon cancer? Q6: What are the different stages and treatments for liver cancer?
Surgery eligibility	Q7: Who is appropriate for surgery for pancreatic cancer? Q8: Who is appropriate for surgery for colon cancer? Q9: Who is appropriate for surgery for liver cancer?
Surgery risks	Q13: What are the risks of surgery to remove my pancreatic cancer? Q14: What are the risks of surgery to remove my colon cancer? Q15: What are the risks of surgery to remove my liver cancer?
General postoperative recovery	Q10: How long is the recovery from pancreatic cancer surgery? Q11: How long is the recovery from colon cancer surgery? Q12: How long is the recovery from liver cancer surgery? Q16: Will I need an ostomy after surgery to remove my colon cancer? Q17: How long will I be in the hospital after surgery for cancer in my belly? Q18: How long after surgery for cancer in my belly can I exercise? Q19: How long will it take to recover from surgery for cancer in my belly? Q20: Should I stay close to the hospital in a hotel or Airbnb after I’m discharged from surgery for cancer in my belly? Q21: How long after surgery for cancer in my belly can I do chores around the house?
Adverse outcomes	Q22: I just had surgery for cancer in my belly, and my incision is painful. What do I do? Q23: I just had surgery for cancer in my belly, and I am still in some pain. Is there anything else I can take for the pain? Q24: I just had surgery for cancer in my belly, and my incision is starting to hurt more and looks slightly open. What do I do? Q25: I just had surgery for cancer in my belly, and the incision is warm to the touch and draining a yellowish fluid. What do I do? Q26: I just had surgery for cancer in my belly. It hurts when I breathe, and I have a new cough. What do I do? Q27: I just had surgery for cancer in my belly. It now burns when I pee. What do I do? Q28: I am about to have surgery for cancer in my belly. How can I prevent an infection after?

### Outcomes or Data Collection and Variables

An anonymous survey was disseminated to surgical faculty at a single institution using Qualtrics, a secure, web-based survey platform. Eligible participants were board-certified surgeons who had completed fellowship training in surgical oncology or colorectal surgery and were actively practicing at the time of the study. Surgeons were identified through publicly available web-based colorectal surgery and surgical oncology faculty rosters and were invited to participate via an email containing the anonymous Qualtrics link. The Qualtrics platform is commonly used in academic research, as it permits investigators to design surveys, test them for accessibility and functionality, distribute them electronically as a web link or QR code, and export result reports. The final list of questions and ChatGPT-4o–generated responses graded by surgeons is included in Multimedia Appendix 1. On the Qualtrics platform, prior to initiating the survey, surgeons were instructed to grade responses for accuracy, quality, and tangibility on a 5-point Likert scale (1=“poor,” 5=“excellent”). The survey defined accuracy as the medical or social correctness of a response, quality as the extent to which a response is well-written and comprehensive, and tangibility as the degree to which the response provides actionable guidance.

After evaluating ChatGPT responses, surgeon respondents were prompted to self-report demographics, including age, sex, years as a practicing surgeon post training, AI frequency in practice, and prior experience with AI. Furthermore, a free response section permitted surgeons to share thoughts or concerns. Respondents were assessed for comfort with patient-AI use through the question, “If a patient informed you they are using publicly available AI (eg, ChatGPT) for health information, how comfortable would you be with encouraging them to use AI following this survey?” Available answer choices included “very uncomfortable,” “uncomfortable,” “neither comfortable nor uncomfortable,” “comfortable,” and “very comfortable.”

LLM-generated responses were separately graded for readability using the Flesch-Kincaid Reading Grade Level (FKRGL) and Flesch Reading Ease (FRE) formulas through the Readability Statistics tool in Microsoft Word Version 16.105.2 [34]. FRE and FKRGL formulas calculate readability based on the average sentence and word length of a text. The FKRGL scale assesses approximate grade level of a text, with an FKRGL score of 5 corresponding to a US 5th-grade reading level. The FRE scale measures readability from 0, unreadable text, to 100, very easily readable text. Both scales were selected as they are validated tools for grading text readability, and they are commonly used by professionals to evaluate the readability of patient-directed health care information [34].

A post hoc analysis was performed to assess FKRGL, FRE scores, and content similarity for GPT responses under 3 prompting conditions: version 1 represented the response to the original question provided for reference; version 2 consisted of responses to the original question preceded by a prompt to “Answer at a 5th-grade level;” and version 3 comprised responses to questions that were reworded to a 5th-grade reading level by ChatGPT-4o prior to response generation. Four questions were selected for post hoc analysis to provide a focused analysis of question-phrasing and prompting on readability. Questions were selected based on having the highest original FKRGL score within 4 different domains and to ensure representation of each malignancy type. Content similarity was graded by 2 independent graders, CVL and DAS, using a 5-point Likert scale (1=not similar, 5=very similar).

### Statistical Analysis

Respondent answers were collected and analyzed in aggregate. Descriptive statistics for categorical variables were reported as frequencies with percentages; continuous variables were reported as mean with SD or median with IQR, where appropriate. Analyses were calculated using Microsoft Excel Version 16.95.4; formulas used included =MEDIAN() for median, =AVERAGE() for mean, =STDEV() for SD, and =QUARTILE.INC() to derive the IQR.

## Results

### Overview

Of the 12 eligible surgeons contacted, 7 responded, resulting in a survey response rate of 58.3%. All respondents were academic surgeons at a single institution. The median reported respondent categorical age range was 35‐44 years. Most survey respondents were male (4/7, 57.1%). Respondents had practiced surgery for a median of 7 (IQR 4‐13) years post training. When assessed for frequency of AI use, 1 respondent reported using AI “daily,” 2 reported using AI “weekly,” 3 reported using AI “monthly,” and 1 reported using AI “almost never.”

### Quality of LLM Responses

When asked to evaluate the quality of responses, experts consistently rated answers between “good” and “very good” to “excellent,” with an aggregate mean response rating of 3.54 (SD 0.28). Across all domains (Table 2), questions related to staging and treatment consistently performed worse, receiving an average rating of 3.33 (SD 0.30), while questions about adverse outcomes tended to perform best, receiving an average rating of 3.73 (SD 0.27). Table 2 indicates the median and IQR of respondent grade for each question, with quality scores ranging from 3.00 to 4.50 and IQR ranging from 2.50-3.50 to 3.25-5.00. The question indicating postoperative urinary tract infection (UTI) received the highest median quality score of 4.50 (3.25‐5.00), between “very good” and “excellent.”

**Table 2.** Quality, accuracy, and tangibility scores for GPT-generated responses^a^.

Domain and question ID	Quality score, median (IQR)	Accuracy score, median (IQR)	Tangibility score, median (IQR)
Signs and symptoms			
Q1	4 (3.5‐4)	4 (4‐4.5)	4 (4‐4.5)
Q2	3 (3-4)	4 (3.5‐4.5)	4 (3-4)
Q3	3 (2.5‐4)	3 (2.5‐4)	3 (3‐3.5)
Stages and treatment			
Q4	3 (2.5‐4.5)	4 (2.5‐4.5)	4 (2.5‐4.5)
Q5	4 (3-4)	4 (3-4)	4 (3.5‐4)
Q6	3 (2.5‐3.5)	3 (2.5‐3.5)	3 (2.5‐3.5)
Surgery eligibility			
Q7	4 (2.5‐4)	4 (2.5‐4)	4 (3.5‐4)
Q8	4 (3.5‐4)	4 (3.5‐4)	4 (3.5‐4)
Q9	4 (4-4)	4 (3.5‐4)	4 (4-4)
General postoperative recovery			
Q10	4 (3-4)	4 (2.5‐4)	4 (3-4)
Q11	3 (2.5‐4)	3 (2.5‐3.5)	3 (2.5‐3.5)
Q12	4 (2.5‐4)	3 (2.5‐3.5)	3 (2.5‐4)
Surgery risks			
Q13	4 (3-4)	4 (3-4)	3 (3-4)
Q14	4 (3.5‐4)	4 (3.5‐4)	4 (3.5‐4)
Q15	3 (3‐3.5)	3 (3‐3.5)	3 (3‐3.5)
General postoperative recovery			
Q16	4 (3-4)	4 (3.5‐4)	4 (3.5‐4)
Q17	3 (2.5‐4)	4 (3.5‐4)	4 (2.5‐4)
Q18	4 (3.5‐4.5)	4 (3.5‐4.5)	4 (3.5‐4)
Q19	4 (3.5‐4)	4 (3.5‐4)	4 (3-4)
Q20	4 (3.5‐4)	4 (3-4)	4 (3-4)
Q21	4 (3-4)	4 (3-4)	4 (3‐4.5)
Adverse outcomes			
Q22	3.5 (3-4)	3.5 (3-4)	3.5 (3-4)
Q23	3.5 (3-4)	4 (3.25‐4)	4 (3.25‐4)
Q24	4 (3.25‐4.75)	4.5 (3.25‐5)	4.5 (3.25‐5)
Q25	4 (3.25‐4)	4 (3.25‐4)	4 (3.25‐4)
Q26	4 (3.25‐4.75)	4.5 (3.25‐5)	4.5 (3.25‐5)
Q27	4.5 (3.25‐5)	4.5 (3.25‐5)	4.5 (3.25‐5)
Q28	4 (4-4)	4 (3.25‐4.75)	4.5 (3.25‐5)

a Accuracy is defined as how “medically or socially accurate” a response is; quality as how “well-written and comprehensive” a response is; and tangibility as how “actionable” a response is.

### Accuracy of LLM Responses

Similar to quality, when asked to evaluate the accuracy of responses, experts generally rated responses between “good” and “very good” to “excellent,” with an aggregate mean response rating of 3.57 (SD 0.34). Across all domains, questions related to staging and treatment performed the worst, receiving an average rating of 3.29 (SD 0.38). Conversely, questions about adverse outcomes consistently performed best, receiving an average rating of 3.83 (SD 0.24). Median response accuracy ratings ranged from 3.00 to 4.50 with IQRs ranging from 2.50-3.50 to 3.25-5.00. Questions regarding postoperative wound dehiscence (Q24), pulmonary embolism (Q26), and UTI management (Q27) received the highest median accuracy grading of 4.50 (IQR 3.25-5.00).

### Tangibility of LLM Responses

When asked to evaluate the tangibility, or how “actionable” a response was, experts likewise consistently rated responses between “good” and “very good” to “excellent,” with an aggregate mean response rating of 3.62 (SD 0.29). Across all domains, questions pertaining to staging and treatment performed the worst, receiving the lowest mean tangibility score of 3.47 (SD 0.30), while questions about adverse outcomes performed best, receiving an average rating of 3.86 (SD 0.28). The median response ratings ranged from 3.00 to 4.50 with IQR scores ranging from 2.50-3.50 to 3.25-5.00. Questions regarding postoperative wound dehiscence (Q24), pulmonary embolism (Q26), UTI management (Q27), and infection prevention (Q28) received the highest median tangibility grading of 4.50 (IQR 3.25-5.00).

### Readability of LLM Responses

When assessing readability (Table 3), ChatGPT-4o–generated responses read at an average FKRGL of 14.51 (SD 1.86), requiring some level of college education for adequate comprehension. Response FKRGL scores ranged from 10.8 to 18.1. A question regarding wound dehiscence received the lowest grade score, 10.8, while a question regarding pancreatic cancer surgery candidacy received the highest score, 18.1. The mean FRE score of ChatGPT-4o–generated responses was 28.8 (SD 9.87), corresponding to a college graduate reading level and indicating low readability. Response FRE scores ranged from 11.7 to 48.0. A question regarding colon cancer surgery recovery had the worst readability, with an FRE score of 11.7. As with FKRGL grading, a question regarding wound dehiscence had the highest ease of readability with an FRE score of 48.0.

**Table 3.** Readability of GPT-generated responses. Readability is represented as FKRGL^a^ and FRE^b^ scores.

ID and question	FKRGL score (US-grade reading level)	FRE score	Estimated FRE US-grade level [34]
Q1: What are the signs and symptoms of pancreatic cancer?	12.9	36.8	13‐16
Q2: What are the signs and symptoms of colon cancer?	12.2	41.3	13‐16
Q3: What are the signs and symptoms of liver cancer?	11.8	41.2	13‐16
Q4: What are the different stages and treatments for pancreatic cancer?	13.4	28.6	College graduate
Q5: What are the different stages and treatments for colon cancer?	15.1	16.4	College graduate
Q6: What are the different stages and treatments for liver cancer?	14.5	17.8	College graduate
Q7: Who is appropriate for surgery for pancreatic cancer?	18.1	20.6	College graduate
Q8: Who is appropriate for surgery for colon cancer?	18.0	17.4	College graduate
Q9: Who is appropriate for surgery for liver cancer?	16.6	21.5	College graduate
Q10: How long is the recovery from pancreatic cancer surgery?	16.1	20.0	College graduate
Q11: How long is the recovery from colon cancer surgery?	16.2	11.7	College graduate
Q12: How long is the recovery from liver cancer surgery?	14.5	17.4	College graduate
Q13. What are the risks of surgery to remove my pancreatic cancer?	14.8	20.7	College graduate
Q14. What are the risks of surgery to remove my colon cancer?	14.2	22.8	College graduate
Q15. What are the risks of surgery to remove my liver cancer?	14.9	15.6	College graduate
Q16. Will I need an ostomy after surgery to remove my colon cancer?	14.2	32.9	13‐16
Q17: How long will I be in the hospital after surgery for cancer in my belly?	14.7	36.2	13‐16
Q18: How long after surgery for cancer in my belly can I exercise?	15.4	26.5	College graduate
Q19: How long will it take to recover from surgery for cancer in my belly?	14.1	31.8	13‐16
Q20: Should I stay close to the hospital in a hotel or Airbnb after I’m discharged from surgery for cancer in my belly?	17.0	32.7	13‐16
Q21: How long after surgery for cancer in my belly can I do chores around the house?	15.3	33.9	13‐16
Q22: I just had surgery for cancer in my belly, and my incision is painful. What do I do?	12.1	43.6	13‐16
Q23: I just had surgery for cancer in my belly, and I am still in some pain. Is there anything else I can take for the pain?	12.7	36.6	13‐16
Q24: I just had surgery for cancer in my belly, and my incision is starting to hurt more and looks slightly open. What do I do?	10.8	48.0	13‐16
Q25: I just had surgery for cancer in my belly, and the incision is warm to the touch and draining a yellowish fluid. What do I do?	13.6	40.0	13‐16
Q26: I just had surgery for cancer in my belly. It hurts when I breathe, and I have a new cough. What do I do?	16.2	27.4	College graduate
Q27: I just had surgery for cancer in my belly. It now burns when I pee. What do I do?	15.0	30.1	13‐16
Q28: I am about to have surgery for cancer in my belly. How can I prevent an infection after?	12.0	36.1	13‐16

a FKRGL: Flesch-Kincaid Reading Grade Level.

b FRE: Flesch Reading Ease.

Four questions were selected for post hoc analysis shown in Table 4 (Q1, Q7, Q11, and Q15). These questions had an original median FKRGL score of 15.6 (IQR 14.4-16.7; range 12.9‐18.1) and FRE score of 18.1 (IQR 14.6-24.7; range: 11.7‐36.8). When GPT-4o was queried to respond to select questions to the level of a 5th-grade reader, the median FKRGL score decreased to 7.1 (IQR 6.1-8.3; range: 5.9‐9.0) and FRE increased to 73.8 (IQR 66.6-79.3; range: 60.1‐80.9). Two independent graders (CVL and DAS) found responses to result in a mean content similarity of 3.88 (SD 0.25) in comparison to the original response. Responses to questions that were rephrased by GPT, with prompting to query at a 5th-grade reading level, resulted in a median FKRGL score of 14.5 (IQR 13.2-15.4; range: 11.6 to 15.8), a median FRE score of 32.0 (IQR 27.0-37.7; range: 21.4‐45.0), and a mean content similarity of 4.63 (SD 0.25) to original responses. The raters had identical scores for 50% (4/8) of responses, with the other 4 responses differing by 1 on the 5-point Likert scale.

**Table 4.** FKRGL^a^ and FRE^b^ scores for select questions (V1), questions prompted to respond at the 5th-grade level (V2), and questions rephrased by GPT to be asked at the 5th-grade level (V3).

Question and versions	FKRGL score	FRE score	Content similarity, mean (SD)
Q1			
V1: What are the signs and symptoms of pancreatic cancer?	12.9	36.8	Reference
V2: Answer at a 5th-grade level: What are the signs and symptoms of pancreatic cancer?	6.1	80.9	4.0 (0)
V3: What are the warning signs of pancreatic cancer and how might someone feel if they have it?	11.6	45.0	4.5 (0.71)
Q7			
V1: Who is appropriate for surgery for pancreatic cancer?	18.1	20.6	Reference
V2: Answer at a 5th-grade level: Who is appropriate for surgery for pancreatic cancer?	9.0	60.1	4.0 (0)
V3: Who can have surgery to treat pancreatic cancer?	15.3	28.8	5.0 (0)
Q11			
V1: How long is the recovery from colon cancer surgery?	16.2	11.7	Reference
V2: Answer at a 5th-grade level: How long is the recovery from colon cancer surgery?	5.9	78.8	3.5 (0.71)
V3: How long does it take to feel better after colon cancer surgery?	15.8	21.4	4.5 (0.71)
Q15			
V1: What are the risks of surgery to remove my liver cancer?	14.9	15.6	Reference
V2: Answer at a 5th-grade level: What are the risks of surgery to remove my liver cancer?	8.1	68.8	4.0 (0)
V3: What could go wrong if I have surgery to take out my liver cancer?	13.7	35.2	4.5 (0.71)

a FKRGL: Flesch-Kincaid Reading Grade Level.

b FRE: Flesch Reading Ease.

### Qualitative Feedback

Numerous inaccuracies within GPT-generated responses were detected by a surgeon-expert concerning general disease information and postoperative recovery. The following feedback has been modified for clarity but maintains the original intent. In Q2 (signs and symptoms of colon cancer), rectal bleeding was mistakenly described as a systemic symptom, while it is a local symptom that may lead to secondary systemic symptoms, including fatigue due to anemia. For Q10 (pancreatic cancer surgery recovery), “light activities,” which are often defined as walking or activities of daily living in the surgical setting, were resumed while a patient was admitted, instead of the written 6 to 12 weeks following discharge. Likewise, for Q11 (colon cancer surgery recovery), certain “light activities” could be resumed sooner. For Q12 (liver cancer surgery recovery), the mention of major hepatectomy as treatment was notably absent.

Regarding quality, numerous content gaps were noted. For Q3 (signs and symptoms of liver cancer), the response described chronic liver disease symptoms; these are common for patients with primary liver cancers but less frequent in the setting of secondary liver cancers (ie, colorectal cancer with liver metastases). Regarding Q4 (stages and treatments of pancreatic cancer), genetic testing should be included when discussing targeted therapies. For Q7 (pancreatic cancer surgery eligibility), discussion of the biology of resectability, which is accounted for by tumor markers such as Ca 19‐9, was notably absent. Regarding Q20 (staying near the hospital following discharge), while listed, it is not emphasized that staying nearby is unnecessary unless the patient lives far away. Furthermore, the question could be enhanced by including discussion of local housing options with case management or a social worker. For Q25 (postoperative infection), concern for dehiscence is not explicitly stated, and the volume of drainage should be addressed sooner, as high volume may indicate dehiscence.

### Provider Recommendations for GPT as a Patient Resource

When assessed on their comfort level with patients using publicly available AI for health information, 57.1% (4/7) of providers reported being “comfortable,” 14.3% (1/7) reported being “neither comfortable nor uncomfortable,” 14.3% (1/7) reported being “uncomfortable,” and 14.3% (1/7) reported being “very uncomfortable.” Regarding provider discomfort, when asked for questions or concerns pertaining to the study, 1 respondent reported “The answers should be designed for a lower health literacy level.” Another physician expressed concern over direct patient use of ChatGPT, primarily citing lack of supervision and noting “health is not something you want to leave up to a robot. There will always be intricacies that cannot be understood by AI.”

## Discussion

### Principal Findings

This study is among the first to evaluate ChatGPT-4o as a patient information resource for individuals preparing for or recovering from surgery for abdominal malignancies [20,35]. As patient self-use of LLMs for medical information is increasing [14], it is essential to assess the content quality, safety, and comprehensibility of GPT-generated responses. Through gaining a deeper understanding of the strengths and weaknesses present within LLMs, providers may help patients be aware of such options and help them navigate the use of these sources. The current study’s results indicate that ChatGPT-4o may serve as a useful patient information resource, with most responses rated from “good” to “very good to excellent” in quality, accuracy, and tangibility. Notably, the lowest rated responses received a median score of 3.0, corresponding to a “good” rating, whereas the highest rated responses received a score of 4.50, corresponding to a rating between “very good” and “excellent.” However, there is still room for improvement in generated responses prior to the endorsement of ChatGPT as a “gold-standard” patient resource. While most providers were “comfortable” having patients use publicly available AI for health information, 42.9% (3/7) of providers did not report feeling “comfortable” having patients use publicly available AI for health information, with 2 reported being “uncomfortable” or “very uncomfortable.” Physicians cited concerns regarding patient use of ChatGPT, noting poor response comprehensibility and lack of supervision, factors likely contributing to their lack of comfort in patient use of ChatGPT. Moreover, this study raises concerns about the comprehensibility of the generated responses, as elevated FKGRL scores indicate that many require a postsecondary reading level for adequate understanding. Physicians should be aware that, in the context of patient use of LLMs for medical information, patients would benefit from instructions for use and monitoring for potential ChatGPT-derived misconceptions.

Information is scarce regarding the safety and accuracy of ChatGPT-generated responses in the perioperative setting for abdominal malignancies. Given the complex biological mechanisms and therapeutic management of gastrointestinal malignancies, it is critical to evaluate the quality of ChatGPT-generated content. The presented data suggest that, although ChatGPT responses averaged as “good” or “very good,” scores were highly question- and domain-dependent. Given its high overall ratings, ChatGPT may serve as an advantageous tool for patients to develop a baseline knowledge of their disease prior to clinical encounters. However, numerous inaccuracies and content gaps were identified within responses. This is congruent with past work assessing ChatGPT’s use for thoracic surgery, where most responses likewise ranged from “good” to “very good,” minor inaccuracies were identified in each answer, and certain domains performed better than others [32]. Regarding abdominal malignancies, questions concerning staging and treatment received the lowest mean accuracy and quality scores. As such, providers should be encouraged to assess potential disease misconceptions that patients using ChatGPT may have and ensure they distribute comprehensive general disease information. Interestingly, ChatGPT-4o excelled in answering questions pertaining to adverse outcomes following surgery. As such, ChatGPT may help guide patients seeking proper management for postoperative complications.

The present study suggests that patient information regarding abdominal malignancies presented by ChatGPT-4o may produce material that is poorly comprehensible for many of the intended population due to requirements of high health literacy and education level. One surgeon expressed that responses should be written for a lower health literacy level. This is consistent with the findings of a high grade level requirement for adequate comprehensibility (FKRGL score), averaging a grade level of 14.5. An FKRGL of 14.5 represents a reading level requiring some level of college education. Current recommendations suggest that patient resources should be tailored to a 5th-grade reading level for accessibility [36,37].

Readability as a limitation of ChatGPT has been previously reported in the literature [15,22]. Past work regarding cervical spine surgery likewise noted high FKRGL scores to limit ChatGPT-3.5’s use as a patient resource. After prompting ChatGPT to provide answers at a 6th-grade reading level, answers decreased from a grade level of 13.5 to 11.2, though remaining persistently elevated. Notably, the present study used similar techniques that successfully produced responses at a lower reading level with ChatGPT-4o. For select questions, the median FKRGL score prior to rephrasing or prompting was 15.6 (IQR 14.4-16.7). Remarkable improvement was noted upon prompting GPT to respond to the level of a 5th-grade reader, decreasing the median FKRGL score, or US grade level, to 7.1 (IQR 6.1-8.3). This work suggests that improvements within ChatGPT-4o may allow for more comprehensible responses, given appropriate prompting. Notably, there was a less meaningful drop in median FKRGL score, from 15.6 (IQR 14.4-16.7) to 14.5 (IQR 13.2-15.4), when ChatGPT was used to *rephrase* questions to be asked at the reading level of a 5th grader. Prior to modification, the median FRE score for select questions was 18.1 (IQR 14.6-24.7). Consistent with FKRGL trends, prompting questions resulted in a more substantial increase in FRE score (median FRE 73.8, IQR 66.6-79.3), indicating markedly improved readability, compared with rephrasing (median FRE 32, IQR 27.0-37.7). This suggests that explicitly requesting ChatGPT to produce responses at a lower level may be more effective in improving readability than adjusting question phrasing. Although contents similar to original questions were better for the latter group than the former (4.63/5 versus 3.88/5), most key concepts were retained within both groups. As such, prompting ChatGPT to answer at a lower grade level may improve readability without significantly sacrificing content. Therefore, providers should be encouraged to assess patient use of LLMs for medical questions and provide patients with a menu for how to prompt ChatGPT to answer at an appropriate grade level if relevant.

While comprehensibility without prompting educational level can be a limitation for the intended patient population, ChatGPT may serve as a useful tool for providers and trainees. Past work in public health has found AI chatbots to be a useful educational tool for medical students in answering complex medical questions [38]. Within the present study, questions 1 to 9 pertain to “signs and symptoms,” “stages and treatment,” and “surgery eligibility”; these questions may be asked by clinicians or learners. While patient readability was limited by a high mean grade level required, the ratings typically ranged from “good” to “very good” in quality, accuracy, and tangibility. This suggests that ChatGPT-4o can serve as a useful resource for physicians and medical trainees, given a higher health literacy than the general population. To further evaluate the use and comprehensibility of ChatGPT as a patient resource, future investigations should involve patient perspectives.

This study has several limitations. First, the small sample size (n=7) of surgical oncologists grading the responses substantially limits statistical power and the reliability of the findings. The single-institutional nature of the study further limits generalizability, as physician responses may reflect regional practice patterns and institutional biases. Future validation should evaluate larger, multi-institutional cohorts to confirm reproducibility and evaluate external validity. Second, the survey incorporated subjective assessments which may limit reproducibility, as concepts graded, such as “quality,” “accuracy,” and “tangibility,” are abstract. To enhance reproducibility, standardized definitions of these domains were included on each page of the survey. Third, questions may not be well representative of patient language. Although questions were obtained from hospital websites and piloted with residents to improve alignment with patient phrasing, they may not encompass the full spectrum of patient inquiries nor the variability of patients’ health literacy. As only 28 questions were assessed across 3 malignancies, the nature of the questions is limited in scope and may not represent all questions patients may ask pertaining to their diagnosed malignancy. Moreover, questions are broad, pertaining to “colon,” “pancreas,” “liver,” or “belly” cancers, without specifying types and stages.

### Conclusions

This preliminary study indicates that, while publicly accessible ChatGPT may serve as a useful patient resource, its use as an unsupervised source of information for patients with abdominal malignancies has distinct limitations. Providers should be aware that many of their patients are accessing ChatGPT and recognize that developing an understanding of its strengths and limitations can help them guide their patients to enable its best use. Inaccuracies, gaps in information, and poor readability were identified in ChatGPT-generated content, suggesting patients may benefit from physician guidance. Providers should be prepared to properly support their patients reporting ChatGPT use by counseling techniques such as prompting questions to tailor responses to their educational level. The data herein indicate that this is critical for the interpretation of the information by patients, as without this guidance, the answers are directed to an educational level of college or above.

## Supplementary material

10.2196/81374 Multimedia Appendix 1 Distributed survey with GPT-generated responses.

## Abbreviations

AI: artificial intelligence
FKRGL: Flesch-Kincaid Reading Grade Level
FRE: Flesch Reading Ease
LLM: large language model
UTI: urinary tract infection

## References

[1] ChatGPT.

[2] Guinness H How does ChatGPT work?. Zapier.

[3] Bubeck S, Chandrasekaran V, Eldan R, Gehrke J, Horvitz E, Kamar E (2023). Sparks of artificial general intelligence: early experiments with GPT-4. arXiv.

[4] Kung TH, Cheatham M, Medenilla A (2023). Performance of ChatGPT on USMLE: potential for AI-assisted medical education using large language models. PLOS Digit Health.

[5] Gilson A, Safranek CW, Huang T (2023). How does ChatGPT perform on the United States Medical Licensing Examination (USMLE)? The implications of large language models for medical education and knowledge assessment. JMIR Med Educ.

[6] Gupta R, Herzog I, Park JB (2023). Performance of ChatGPT on the plastic surgery inservice training examination. Aesthet Surg J.

[7] Hoch CC, Wollenberg B, Lüers JC (2023). ChatGPT’s quiz skills in different otolaryngology subspecialties: an analysis of 2576 single-choice and multiple-choice board certification preparation questions. Eur Arch Otorhinolaryngol.

[8] Mihalache A, Huang RS, Popovic MM, Muni RH (2023). Performance of an upgraded artificial intelligence chatbot for ophthalmic knowledge assessment. JAMA Ophthalmol.

[9] Cabral S, Restrepo D, Kanjee Z (2024). Clinical reasoning of a generative artificial intelligence model compared with physicians. JAMA Intern Med.

[10] Tan S, Xin X, Wu D (2024). ChatGPT in medicine: prospects and challenges: a review article. Int J Surg.

[11] Liu HY, Alessandri Bonetti M, De Lorenzi F, Gimbel ML, Nguyen VT, Egro FM (2024). Consulting the digital doctor: Google versus ChatGPT as sources of information on breast implant-associated anaplastic large cell lymphoma and breast implant illness. Aesthetic Plast Surg.

[12] Bergmo TS, Sandsdalen V, Manskow US, Småbrekke L, Waaseth M (2023). Internet use for obtaining medicine information: cross-sectional survey. JMIR Form Res.

[13] Ramli R, Jambor MA, Kong CY (2023). Dr Google - assessing the reliability and readability of information on general surgical procedures found via search engines. ANZ J Surg.

[14] Ayre J, Cvejic E, McCaffery KJ (2025). Use of ChatGPT to obtain health information in Australia, 2024: insights from a nationally representative survey. Med J Aust.

[15] Shen SA, Perez-Heydrich CA, Xie DX, Nellis JC (2024). ChatGPT vs. web search for patient questions: what does ChatGPT do better?. Eur Arch Otorhinolaryngol.

[16] Artioli E, Veronesi F, Mazzotti A (2024). Assessing ChatGPT responses to common patient questions regarding total ankle arthroplasty. J Exp Orthop.

[17] Gajjar AA, Kumar RP, Paliwoda ED (2024). Usefulness and accuracy of artificial intelligence chatbot responses to patient questions for neurosurgical procedures. Neurosurgery.

[18] Samaan JS, Yeo YH, Rajeev N (2023). Assessing the accuracy of responses by the language model ChatGPT to questions regarding bariatric surgery. Obes Surg.

[19] Yeo YH, Samaan JS, Ng WH (2023). Assessing the performance of ChatGPT in answering questions regarding cirrhosis and hepatocellular carcinoma. Clin Mol Hepatol.

[20] Rydzewski NR, Dinakaran D, Zhao SG (2024). Comparative evaluation of LLMs in clinical oncology. NEJM AI.

[21] Lee TC, Staller K, Botoman V, Pathipati MP, Varma S, Kuo B (2023). ChatGPT answers common patient questions about colonoscopy. Gastroenterology.

[22] Subramanian T, Araghi K, Amen TB (2024). Chat generative pretraining transformer answers patient-focused questions in cervical spine surgery. Clin Spine Surg.

[23] Al-Dujaili Z, Omari S, Pillai J, Al Faraj A (2023). Assessing the accuracy and consistency of ChatGPT in clinical pharmacy management: a preliminary analysis with clinical pharmacy experts worldwide. Res Social Adm Pharm.

[24] Arnold M, Abnet CC, Neale RE (2020). Global burden of 5 major types of gastrointestinal cancer. Gastroenterology.

[25] (2025). Pancreatic cancer questions to ask the healthcare team. Pancreatic Cancer Action Network.

[26] (2025). Colon cancer FAQs. Moffitt Cancer Center.

[27] (2025). Frequently asked questions. Mount Sinai Tisch Cancer Center.

[28] (2025). Liver cancer – frequently asked questions. Pelican Cancer Foundation.

[29] (2024). Questions to ask about pancreatic cancer. American Cancer Society.

[30] (2025). Questions to ask about liver cancer. American Cancer Society.

[31] (2025). Frequently asked questions. Hirshberg Foundation for Pancreatic Cancer Research.

[32] Ferrari-Light D, Merritt RE, D’Souza D (2025). Evaluating ChatGPT as a patient resource for frequently asked questions about lung cancer surgery-a pilot study. J Thorac Cardiovasc Surg.

[33] (2026). ChatGPT — release notes. OpenAI.

[34] Jindal P, MacDermid JC (2017). Assessing reading levels of health information: uses and limitations of flesch formula. Educ Health (Abingdon).

[35] Munir MM, Endo Y, Ejaz A, Dillhoff M, Cloyd JM, Pawlik TM (2024). Online artificial intelligence platforms and their applicability to gastrointestinal surgical operations. J Gastrointest Surg.

[36] (2024). AHRQ health literacy universal precautions toolkit. https://www.ahrq.gov/sites/default/files/wysiwyg/health-literacy/3rd-edition-toolkit/health-literacy-toolkit-third-edition.pdf.

[37] Stossel LM, Segar N, Gliatto P, Fallar R, Karani R (2012). Readability of patient education materials available at the point of care. J Gen Intern Med.

[38] Baglivo F, De Angelis L, Casigliani V, Arzilli G, Privitera GP, Rizzo C (2023). Exploring the possible use of AI chatbots in public health education: feasibility study. JMIR Med Educ.

