# Supplementary material for: Physician Perspectives on ChatGPT-4o as a Patient Resource for Abdominal Cancer Surgeries: Cross-Sectional Survey
Source: JMIR Perioper Med. 2026 May 4;9:e81374. doi: 10.2196/81374 (PMC13138707; doi:10.2196/81374)
Supplement: Multimedia Appendix 1 [file periop-v9-e81374-s001.docx]

Appendix A: Distributed Survey with GPT-Generated Responses

*Question 1: What are the signs and symptoms of pancreatic cancer?*

Response 1: Pancreatic cancer can be challenging to detect early, as symptoms often appear only in advanced stages. Common signs include abdominal pain that may radiate to the back, unintended weight loss, and changes in digestion, such as greasy or pale stools due to poor fat absorption. Jaundice, characterized by yellowing of the skin and eyes, dark urine, and pale stools, may occur if the tumor blocks the bile duct. Other symptoms include nausea, vomiting, and a loss of appetite or early satiety, which can contribute to significant weight loss. Additionally, individuals may experience general symptoms like fatigue, unexplained new or worsening diabetes, and blood clots, such as deep vein thrombosis. Itchy skin and abdominal swelling from fluid accumulation (ascites) can also occur. If you notice any persistent symptoms like jaundice, unexplained weight loss, or new-onset diabetes, it’s crucial to consult a healthcare provider promptly. Early detection and diagnosis can significantly impact treatment options and outcomes.

*Question 2: What are the signs and symptoms of colon cancer?*

Response 2: Colon cancer can present with a range of signs and symptoms, often depending on the size and location of the tumor within the colon. Early stages may not cause noticeable symptoms, but as the disease progresses, common warning signs include changes in bowel habits, such as persistent diarrhea, constipation, or a feeling of incomplete evacuation. Narrow or ribbon-like stools can also occur, especially if the tumor is obstructing the colon. Other digestive symptoms may include abdominal discomfort, cramping, bloating, or gas. More systemic symptoms include rectal bleeding, blood in the stool (which can be bright red or dark), and unexplained weight loss. Fatigue and weakness are also common due to anemia caused by chronic blood loss from the tumor. In some cases, individuals may experience no symptoms until the disease is advanced, which is why routine screening is crucial for early detection. If you notice persistent changes in bowel habits, blood in your stool, or unexplained fatigue, it’s important to seek medical evaluation promptly. Early diagnosis can significantly improve outcomes.

*Question 3: What are the signs and symptoms of liver cancer?*

Response 3: Liver cancer often does not cause symptoms in its early stages, but as the disease progresses, various signs may emerge. Common symptoms include unexplained weight loss, loss of appetite, and persistent fatigue. Abdominal pain or discomfort, particularly on the right side or near the right shoulder blade, can occur as the liver enlarges. Swelling or bloating in the abdomen (ascites) may also be noticeable, often due to fluid accumulation. Other signs include jaundice, which presents as yellowing of the skin and eyes, along with dark urine and pale stools, indicating a buildup of bilirubin. Nausea, vomiting, and general weakness are also common symptoms. Some individuals may experience itching or feel a lump in the upper right abdomen. If you have a history of liver disease, such as cirrhosis or hepatitis, and notice any of these symptoms, it is important to seek medical attention promptly. Early detection is critical, as liver cancer often progresses rapidly improve outcomes.

*Question 4: What are the different stages and treatments for pancreatic cancer?*

Response 4: Pancreatic cancer is categorized into stages based on the tumor’s size, location, and whether it has spread. Stage 1 is confined to the pancreas, with surgical removal often being an option. Stage 2 indicates limited spread to nearby tissues or lymph nodes, where surgery may still be possible, often combined with chemotherapy or radiation to improve outcomes. Stage 3 involves cancer that has invaded nearby major blood vessels or more lymph nodes, making surgery difficult. Treatment typically focuses on chemotherapy and radiation to shrink the tumor and manage symptoms. Stage 4 is advanced pancreatic cancer with distant metastases, where treatment is primarily palliative, focusing on managing symptoms and slowing progression with chemotherapy or targeted therapies. Treatment depends on the stage. Surgery, such as the Whipple procedure, is the primary option for early stages. When surgery isn’t feasible, chemotherapy (e.g., gemcitabine, FOLFIRINOX) and radiation are used to manage the disease. Targeted therapies and immunotherapies, though less commonly used, may be considered in specific cases. Palliative care, including pain management and nutritional support, is crucial across all stages to improve quality of life.

*Question 5: What are the different stages and treatments for colon cancer?*

Response 5: Colon cancer is staged from Stage 0 (in situ) to Stage 4 (metastatic). Stage 0 involves localized, non-invasive cancer that is usually treated with polyp removal or localized excision during a colonoscopy. Stage 1 indicates that the cancer has invaded the colon wall but has not spread to lymph nodes, typically treated with surgical resection. Stage 2 involves deeper invasion into or through the colon wall, sometimes requiring surgery with or without chemotherapy. Stage 3 includes lymph node involvement, and treatment often involves surgery followed by chemotherapy. Stage 4 indicates metastasis to distant organs, such as the liver or lungs, requiring systemic therapies like chemotherapy, targeted therapies, or immunotherapy. Treatment varies by stage. Surgery is the primary treatment for localized colon cancer, with laparoscopic or open procedures depending on the extent. Adjuvant chemotherapy, often with drugs like FOLFOX (5-FU, leucovorin, oxaliplatin), is used to lower recurrence risk in later stages. In metastatic cases, systemic therapies (e.g., bevacizumab, cetuximab) or regional treatments like liver-directed therapies are common. Immunotherapy, including checkpoint inhibitors, may be effective for certain genetic subtypes like microsatellite instability-high (MSI-H) tumors.

*Question 6: What are the different stages and treatments for liver cancer?*

Response 6: Liver cancer is classified by stages, often using systems like the Barcelona Clinic Liver Cancer (BCLC) staging system. Early-stage liver cancer (BCLC Stage A) involves a single tumor or limited tumors that haven’t spread. Curative treatments like surgical resection, liver transplantation, or localized therapies (e.g., radiofrequency ablation) are often used. Intermediate-stage cancer (BCLC Stage B) includes larger or multiple tumors confined to the liver, treated with transarterial chemoembolization (TACE) or other locoregional therapies. Advanced-stage cancer (BCLC Stage C) involves spread to blood vessels or other organs, treated with systemic therapies. End-stage cancer (BCLC Stage D) focuses on palliative care. Treatment depends on factors like liver function, tumor size, and overall health. Surgical resection or transplantation is ideal for early-stage disease when liver function is intact. If surgery isn’t possible, ablation or TACE can target tumors directly. Systemic therapies, including targeted drugs like sorafenib or lenvatinib, are standard for advanced disease. Recent advancements in immunotherapy, such as checkpoint inhibitors like atezolizumab combined with bevacizumab, have shown promise in treating advanced cases. Symptom management, including pain relief and nutritional support, is integral at all stages.

*Question 7: Who is appropriate for surgery for pancreatic cancer?*

Response 7: Surgery for pancreatic cancer is typically appropriate for patients whose cancer is confined to the pancreas and has not spread to major blood vessels, distant organs, or lymph nodes. These patients are considered to have "resectable" or sometimes "borderline resectable" tumors, meaning the cancer can be surgically removed with a high chance of success. The overall health of the patient also plays a significant role, as pancreatic surgery, such as the Whipple procedure, is complex and requires a patient to be strong enough to tolerate the operation and recovery. Patients with locally advanced or metastatic pancreatic cancer, where the disease has spread beyond the pancreas, are usually not candidates for surgery and are instead treated with chemotherapy, radiation, or palliative care to manage symptoms and improve quality of life. In some cases, neoadjuvant therapy, which includes chemotherapy or radiation before surgery, may be used to shrink borderline tumors to make surgical removal more feasible.

*Question 8: Who is appropriate for surgery for colon cancer?*

Response 8: Surgery for colon cancer is typically appropriate for individuals diagnosed with localized or resectable colon cancer, meaning the cancer is confined to the colon or nearby lymph nodes and has not spread extensively to distant organs. Candidates for surgery often include those with early-stage (Stage I and II) and some Stage III cancers, where removal of the tumor and surrounding tissue can be curative. In cases of Stage IV colon cancer, surgery may be considered if the cancer has spread to limited areas, such as the liver or lungs, and if removing these metastases could improve survival or alleviate symptoms. Additionally, a patient's overall health, including factors such as age, heart and lung function, and ability to recover from surgery, plays a crucial role in determining eligibility. Those with significant medical conditions that may increase surgical risks might be evaluated for alternative treatments like chemotherapy, radiation, or targeted therapies. Ultimately, the decision for surgery is made by a multidisciplinary team, considering the cancer stage, patient health, and potential benefits of the procedure.

*Question 9: Who is appropriate for surgery for liver cancer?*

Response 9: Surgery for liver cancer is typically appropriate for patients who have a single tumor confined to the liver without significant involvement of major blood vessels or distant spread. Candidates should have good overall liver function, usually measured by the Child-Pugh score, and no severe underlying liver disease, such as advanced cirrhosis. Ideal candidates for surgical resection should also be in good general health to tolerate the procedure and recovery. In cases where the liver function is significantly impaired or multiple tumors are present, a liver transplant may be considered instead, provided the patient meets criteria such as the Milan or UCSF guidelines. However, surgery is generally not suitable for patients with extensive tumor spread, poor liver function, or other significant medical conditions that increase surgical risk.

*Question 10: How long is the recovery from pancreatic cancer surgery?*

Response 10: Recovery from pancreatic cancer surgery, such as the Whipple procedure, typically takes several months due to the complexity of the operation. The hospital stay is usually about 7-14 days, during which time the medical team monitors for complications like infection, delayed gastric emptying, or digestive issues. Once discharged, it may take 6-12 weeks to resume light activities, but full recovery can take up to 6 months or more. During recovery, patients may experience fatigue, weight loss, and digestive changes, as the surgery often impacts the ability to process food and manage blood sugar levels. A specialized diet and support from a nutritionist are often necessary, along with physical therapy to rebuild strength. Regular follow-up appointments and possible adjuvant therapy (chemotherapy or radiation) are part of the recovery process, requiring ongoing adjustments to optimize long-term health.

*Question 11: How long is the recovery from colon cancer surgery?*

Response 11: Recovery from colon cancer surgery depends on the type of procedure and the individual’s overall health. For minimally invasive surgeries, such as laparoscopic or robotic-assisted colectomy, patients are often discharged within 3-5 days and can resume light activities in about 2-4 weeks. Open surgeries may require a longer hospital stay (5-7 days) and recovery period, often taking 6-8 weeks to return to normal activities. Patients may experience temporary changes in bowel habits, fatigue, or abdominal discomfort during recovery. A balanced diet, hydration, and gradual physical activity can aid in the healing process. Full recovery can take several months, and ongoing follow-ups may include chemotherapy for advanced stages or monitoring for recurrence.

*Question 12: How long is the recovery from liver cancer surgery?*

Response 12: Recovery from liver cancer surgery, whether a partial hepatectomy (resection) or liver transplantation, varies depending on the procedure and patient health. After a liver resection, most patients stay in the hospital for 5-10 days. Light activities can often resume within 4-6 weeks, but full recovery may take up to 3-4 months. The liver’s ability to regenerate typically aids in the recovery process. For liver transplantation, recovery is more extensive. Patients remain in the hospital for 1-2 weeks, followed by several months of monitoring to ensure the body accepts the new organ. Immunosuppressive medications are required to prevent rejection, adding complexity to the recovery. Both procedures require lifestyle adjustments, regular follow-ups, and possibly adjuvant treatments to manage long-term outcomes.

*Question 13. What are the risks of surgery to remove my pancreatic cancer?*

Response 13: Surgery to remove pancreatic cancer, such as the Whipple procedure, is complex and carries significant risks. Common risks include bleeding, infection, and delayed gastric emptying, where the stomach takes longer to empty food. Other potential complications include leakage at the surgical connections (anastomoses) between the pancreas, stomach, and intestines, which may require additional interventions. Long-term risks include malabsorption, as the pancreas may no longer produce sufficient digestive enzymes, and diabetes, if enough insulin-producing cells are removed. Additional risks depend on the patient’s overall health and the extent of the surgery. Recovery can be prolonged, and complications like blood clots, pneumonia, or significant weight loss are possible. Careful post-operative monitoring and support from a multidisciplinary team can help manage these risks and improve recovery outcomes.

*Question 14. What are the risks of surgery to remove my colon cancer?*

Response 14: Surgery for colon cancer is generally safe but carries some risks. Common complications include infection, bleeding, and bowel obstruction. Leakage at the site where the colon is reconnected (anastomotic leak) is another potential risk, which may require further surgery. Patients may also experience temporary or permanent changes in bowel habits, including diarrhea, constipation, or urgency. Other risks include damage to nearby organs, blood clots, or delayed wound healing, especially in older adults or those with underlying health conditions. While serious complications are rare, a thorough preoperative evaluation and proper post-operative care can help reduce risks and ensure a smoother recovery.

*Question 15. What are the risks of surgery to remove my liver cancer?*

Response 15: Liver cancer surgery, whether a partial resection or a liver transplant, carries risks primarily due to the complexity of the procedure. Bleeding is a significant concern because the liver has a rich blood supply. Other risks include infection, bile leakage, or damage to surrounding organs. In liver transplant patients, there is also a risk of organ rejection, requiring lifelong immunosuppressive therapy. Post-operative complications can include liver failure, especially if the remaining liver is insufficiently healthy to regenerate. Fatigue, temporary jaundice, or fluid accumulation (ascites) may also occur during recovery. A carefully planned surgical approach and close post-operative monitoring are essential to minimize these risks and support recovery.

*Question 16. Will I need an ostomy after surgery to remove my colon cancer?*

Response 16: Whether you need an ostomy after colon cancer surgery depends on the location and extent of the tumor. For cancers in the lower part of the colon or rectum, particularly near the anus, a temporary or permanent ostomy (colostomy or ileostomy) may be necessary. This is often the case if the surgery involves removing the rectum or if healing at the surgical site requires protection from stool passing through. For cancers in the upper colon, an ostomy is less likely, as surgeons can often reconnect the remaining sections of the colon. Temporary ostomies are sometimes used to allow the bowel to heal and may be reversed in a later surgery. Your surgeon will discuss this possibility with you during preoperative planning, ensuring you understand the need for an ostomy and any associated care.

*Question 17: How long will I be in the hospital after surgery for cancer in my belly?*

Response 17: The length of your hospital stay after surgery for cancer in your abdomen depends on several factors, including the type of cancer, the complexity of the surgery, and your overall health. For minimally invasive procedures, such as laparoscopic or robotic-assisted surgeries, you may only need to stay in the hospital for a few days, typically around two to five days. However, if you undergo a more extensive open surgery, such as a major bowel resection or removal of multiple organs, your stay could be longer, often ranging from a week to two weeks or more. Recovery time also depends on how well your body heals, whether there are any complications, and how soon you can start eating and moving around. Your doctor will monitor your progress and determine when it is safe for you to go home. Once discharged, you may still need follow-up care, physical therapy, or additional treatments, depending on your diagnosis and overall recovery.

*Question 18: How long after surgery for cancer in my belly can I exercise?*

Response 18: The timeline for returning to exercise after abdominal cancer surgery depends on several factors, including the type of surgery, your overall health, and how well your body is healing. Generally, most patients can begin light activities, such as walking, within a few days to promote circulation and prevent complications. However, more strenuous activities, such as lifting weights or engaging in core exercises, may need to be avoided for at least six to eight weeks or longer, depending on your surgeon's recommendations. It's important to follow your doctor's guidance and listen to your body, gradually increasing activity as tolerated. If you experience pain, swelling, or other concerning symptoms, you should stop exercising and consult your healthcare provider.

*Question 19: How long will it take to recover from surgery for cancer in my belly?*

Response 19: Recovery time from surgery for cancer in your abdomen depends on several factors, including the type and extent of the surgery, your overall health, and how well your body heals. Generally, major abdominal surgeries, such as those involving the removal of part of an organ or tumor resection, can take several weeks to months for full recovery. In the first few days after surgery, you may experience pain, fatigue, and limited mobility, with hospital stays typically ranging from a few days to over a week. During the first few weeks at home, you will need to follow dietary restrictions, avoid strenuous activities, and gradually regain strength. Most people can resume normal activities within six to eight weeks, though complete internal healing may take longer. If chemotherapy or radiation is needed after surgery, recovery may be prolonged due to additional side effects. Following your doctor’s post-operative instructions, maintaining good nutrition, and allowing yourself adequate rest will help speed up recovery. Always consult your healthcare provider for specific guidance tailored to your condition.

*Question 20: Should I stay close to the hospital in a hotel or AirBnB after I'm discharged from surgery for cancer in my belly?*

Response 20: Staying close to the hospital in a hotel or Airbnb after your surgery could be a wise decision, especially if your medical team anticipates the need for follow-up care, wound checks, or emergency visits in the days immediately after discharge. Since belly surgery can be major, recovery might come with unexpected complications such as pain, infections, or difficulty moving around, and being near the hospital could provide peace of mind and easier access to care if needed. A hotel may offer convenience with housekeeping and amenities, while an Airbnb might provide a more comfortable, home-like environment where you can rest without the sterile feel of a hospital. Ultimately, your choice should be based on factors like how far your home is from the hospital, the level of support you have from family or caregivers, and your personal comfort. If your home is too far or difficult to return to in case of complications, staying nearby—even for a few days—could be a good precautionary measure.

*Question 21: How long after surgery for cancer in my belly can I do chores around the house?*

Response 21: The timeline for resuming household chores after surgery for cancer in your abdomen depends on the type and extent of the procedure, as well as your overall health and recovery progress. Generally, most patients can begin light activities, such as walking and minimal household tasks, within a few days to a couple of weeks, but heavy lifting, bending, and strenuous chores should be avoided for at least four to six weeks. Your surgeon will provide specific guidelines based on your individual case, including any restrictions on lifting weight or engaging in certain movements that could strain your healing abdomen. It's essential to listen to your body, take breaks when needed, and gradually increase activity as tolerated. If you experience pain, fatigue, or any concerning symptoms, you should consult your doctor before resuming more demanding tasks.

*Question 22: I just surgery for cancer in my belly, and my incision is painful. What do I do?*

Response 22: Managing pain after cancer surgery is important for your recovery. Make sure you're following your doctor's instructions for pain medication, whether it's prescription or over-the-counter. Keeping your incision clean and dry can help prevent infection, which could make the pain worse. Try to rest and avoid straining the area, but also follow any movement guidelines your doctor has given you to prevent complications like blood clots. Applying a cold pack (wrapped in a cloth) near the incision may help reduce swelling, but avoid putting ice directly on the wound. If the pain is severe, worsening, or accompanied by redness, swelling, fever, or drainage from the incision, contact your doctor immediately, as these could be signs of an infection or other complications. Staying hydrated and eating nutritious foods can also support healing and help you feel better overall.

*Question 23: I just had surgery for cancer in my belly, and I am still in some pain. Is there anything else I can take for the pain?*

Response 23: I'm sorry you're going through this. Managing pain after surgery is important for your recovery, and there may be additional options to help you feel more comfortable. If your current medication isn't providing enough relief, you should talk to your doctor about adjusting the dosage or trying a different pain reliever. Depending on your situation, they may recommend stronger prescription painkillers, nerve pain medications, or even certain anti-inflammatory drugs. Non-medication approaches like heat or cold therapy, relaxation techniques, and gentle movement may also help. It's essential to follow your doctor's advice, as some medications could interfere with your healing or have side effects. If the pain is severe or worsening, reach out to your healthcare provider as soon as possible to make sure everything is healing properly.

*Question 24: I just had surgery for cancer in my belly, and my incision is starting to hurt more and looks slightly open. What do I do?*

Response 24: If your incision is starting to hurt more and appears slightly open, it's important to take it seriously. Increased pain and an opening in the incision could indicate an infection or improper healing. You should check for signs of infection, such as redness, swelling, warmth, discharge, or fever. Avoid touching the area with unwashed hands, and keep the incision clean and dry. If you were given specific wound care instructions by your surgeon, follow them closely. However, because worsening pain and an opening in the incision can be concerning, you should contact your doctor or surgeon as soon as possible to have it evaluated. If you experience severe pain, fever, or any signs of infection, seek medical attention immediately.

*Question 25: I just had surgery for cancer in my belly, and the incision is warm to the touch and draining a yellow-ish fluid. What do I do?*

Response 25: It sounds like your incision may be showing signs of infection, especially if it is warm to the touch and draining yellowish fluid. It’s important to monitor for other symptoms such as increased redness, swelling, a foul odor, worsening pain, or fever, as these could indicate a more serious infection. You should contact your surgeon or healthcare provider as soon as possible to discuss your symptoms and determine if you need antibiotics or further medical intervention. In the meantime, keep the area clean and dry, avoid touching it with unwashed hands, and follow any wound care instructions given by your doctor. If you develop a fever or if the drainage becomes excessive or foul-smelling, seek medical attention immediately.

*Question 26: I just had surgery for cancer in my belly. It hurts when I breathe, and I have a new cough. What do I do?*

Response 26: I'm not a doctor, but given that you're experiencing pain when breathing and a new cough after abdominal cancer surgery, you should reach out to your medical team as soon as possible. These symptoms could be related to post-surgical inflammation, irritation from anesthesia, or even something more serious like a lung infection or a pulmonary embolism. In the meantime, try to take slow, deep breaths to prevent lung complications, stay hydrated, and follow any post-op care instructions you were given. However, because breathing issues can sometimes indicate a more urgent problem, it's best to contact your doctor or seek immediate medical attention to ensure everything is healing properly.

*Question 27: I just had surgery for cancer in my belly. It now burns when I pee. What do I do?*

Response 27: Burning during urination after abdominal cancer surgery could be a sign of a urinary tract infection (UTI), irritation from a catheter (if one was used), or inflammation from the surgery itself. It’s important to monitor for other symptoms like fever, cloudy or foul-smelling urine, or increased pain, which could indicate an infection requiring antibiotics. Staying hydrated and drinking plenty of water can help flush out any irritation, but you should contact your doctor as soon as possible to discuss your symptoms. They may want to perform a urine test to check for infection and ensure that there are no complications related to the surgery. If the burning worsens or you experience additional concerning symptoms, seek medical attention promptly.

*Question 28: I am about to have surgery for cancer in my belly. How can I prevent an infection after?*

Response 28: Preventing infection after surgery is crucial for a smooth recovery. Follow your doctor's instructions carefully, including keeping the surgical site clean and dry. Wash your hands thoroughly before touching the area, and avoid submerging it in water until cleared by your doctor. Change dressings as instructed, using only sterile materials. Pay attention to signs of infection, such as increased redness, swelling, warmth, pus, or fever, and report any concerns to your healthcare provider immediately. Eat a nutritious diet rich in protein and vitamins to support healing, and stay hydrated. Avoid smoking and excessive alcohol consumption, as they can slow recovery and increase infection risk. Take prescribed antibiotics as directed, and limit strenuous activities to prevent strain on the surgical site. Finally, make sure to attend all follow-up appointments so your doctor can monitor your healing progress.

Appendix B: Distributed survey for readability analysis with FKRGL scores. Original questions were prompted to be written to the 5^th^ grade reading level and rephrased to query at the level of a 5^th^ grade reading level with GPT-assistance.

*Q1V1: What are the signs and symptoms of pancreatic cancer?*

Pancreatic cancer can be challenging to detect early, as symptoms often appear only in advanced stages. Common signs include abdominal pain that may radiate to the back, unintended weight loss, and changes in digestion, such as greasy or pale stools due to poor fat absorption. Jaundice, characterized by yellowing of the skin and eyes, dark urine, and pale stools, may occur if the tumor blocks the bile duct. Other symptoms include nausea, vomiting, and a loss of appetite or early satiety, which can contribute to significant weight loss. Additionally, individuals may experience general symptoms like fatigue, unexplained new or worsening diabetes, and blood clots, such as deep vein thrombosis. Itchy skin and abdominal swelling from fluid accumulation (ascites) can also occur. If you notice any persistent symptoms like jaundice, unexplained weight loss, or new-onset diabetes, it’s crucial to consult a healthcare provider promptly. Early detection and diagnosis can significantly impact treatment options and outcomes.

*Q1V2. Answer at a 5^th^ grade level: What are the signs and symptoms of pancreatic cancer?*

Pancreatic cancer is a sickness that can be hard to notice at first, but there are some signs to look out for. A person might have pain in their belly or back that doesn’t go away. They might lose weight without trying or not feel hungry at all. Sometimes, their skin or the whites of their eyes can turn yellow, which is called jaundice. People with pancreatic cancer might also feel very tired or sick to their stomach. These symptoms can be caused by other things too, so it’s important to see a doctor if someone doesn’t feel well for a while.

*Q1V3.* *What are the warning signs of pancreatic cancer and how might someone feel if they have it?*

Pancreatic cancer often develops silently, with symptoms that may not appear until the disease is advanced. Some common warning signs include persistent abdominal or back pain, unexplained weight loss, loss of appetite, and jaundice—yellowing of the skin or eyes. People might also experience nausea, changes in stool (such as oily or light-colored stools), and new-onset diabetes. If someone has pancreatic cancer, they might feel increasingly fatigued and weak, with a general sense that something isn’t right. These symptoms can be subtle at first, which is why pancreatic cancer is often difficult to detect early.

*Q7V1: Who is appropriate for surgery for pancreatic cancer?*

Surgery for pancreatic cancer is typically appropriate for patients whose cancer is confined to the pancreas and has not spread to major blood vessels, distant organs, or lymph nodes. These patients are considered to have "resectable" or sometimes "borderline resectable" tumors, meaning the cancer can be surgically removed with a high chance of success. The overall health of the patient also plays a significant role, as pancreatic surgery, such as the Whipple procedure, is complex and requires a patient to be strong enough to tolerate the operation and recovery. Patients with locally advanced or metastatic pancreatic cancer, where the disease has spread beyond the pancreas, are usually not candidates for surgery and are instead treated with chemotherapy, radiation, or palliative care to manage symptoms and improve quality of life. In some cases, neoadjuvant therapy, which includes chemotherapy or radiation before surgery, may be used to shrink borderline tumors to make surgical removal more feasible.

*Q7V2. Answer at a 5^th^ grade level: Who is appropriate for surgery for pancreatic cancer?*

People who are appropriate for surgery for pancreatic cancer are usually those whose cancer is found early and hasn’t spread to other parts of the body. Doctors check if the tumor is in a spot where it can be safely removed. The person also needs to be healthy enough to go through surgery and recover afterward. If the cancer is too big or has spread, surgery might not be the best option. In those cases, doctors may suggest other treatments like chemotherapy or radiation first.

*Q7V3.* *Who can have surgery to treat pancreatic cancer?*

Surgery to treat pancreatic cancer is typically an option for patients whose cancer is detected at an early stage and is confined to the pancreas or nearby areas without having spread extensively. Candidates for surgery generally need to be in good overall health, with organs and systems strong enough to withstand a major operation. The most common surgical procedure, known as the Whipple procedure, is often used when the tumor is located in the head of the pancreas. However, not all patients are eligible for surgery—if the cancer has spread to distant organs or involves major blood vessels that cannot be safely bypassed or removed, surgery may not be recommended. In some cases, chemotherapy or radiation may be used before surgery to shrink the tumor and improve surgical outcomes. A multidisciplinary medical team evaluates each case individually to determine whether surgery is a viable and potentially beneficial option.

*Q11V1. How long is the recovery from colon cancer surgery?*

Recovery from colon cancer surgery depends on the type of procedure and the individual’s overall health. For minimally invasive surgeries, such as laparoscopic or robotic-assisted colectomy, patients are often discharged within 3-5 days and can resume light activities in about 2-4 weeks. Open surgeries may require a longer hospital stay (5-7 days) and recovery period, often taking 6-8 weeks to return to normal activities. Patients may experience temporary changes in bowel habits, fatigue, or abdominal discomfort during recovery. A balanced diet, hydration, and gradual physical activity can aid in the healing process. Full recovery can take several months, and ongoing follow-ups may include chemotherapy for advanced stages or monitoring for recurrence.

Q11V2. *Answer at a 5^th^ grade level: How long is the recovery from colon cancer surgery?*

Recovery from colon cancer surgery usually takes about 6 weeks. During this time, a person needs to rest and take care of their body so it can heal. At first, they might feel tired and have some pain in their belly. They may not be able to eat regular food right away and will need to start with soft or liquid foods. Slowly, they will start to feel better and can do more things each day. Doctors will check on them to make sure everything is healing well. Everyone is a little different, so some people may take a bit longer to feel fully better.

*Q11V3. How long does it take to feel better after colon cancer surgery?*

Recovery after colon cancer surgery varies from person to person, but most people begin to feel significantly better within 6 to 8 weeks. During the first couple of weeks, it's normal to experience fatigue, discomfort, and limited mobility as the body heals from the operation. Full recovery, including regaining strength and returning to normal daily activities, can take several months, especially if additional treatments like chemotherapy are needed. Factors like the type of surgery, the stage of cancer, a person's overall health, and whether there were any complications all influence how quickly someone recovers. It's important to follow medical advice, attend follow-up appointments, and gradually increase physical activity to support the healing process.

*Q 15V1. What are the risks of surgery to remove my liver cancer?*

Liver cancer surgery, whether a partial resection or a liver transplant, carries risks primarily due to the complexity of the procedure. Bleeding is a significant concern because the liver has a rich blood supply. Other risks include infection, bile leakage, or damage to surrounding organs. In liver transplant patients, there is also a risk of organ rejection, requiring lifelong immunosuppressive therapy. Post-operative complications can include liver failure, especially if the remaining liver is insufficiently healthy to regenerate. Fatigue, temporary jaundice, or fluid accumulation (ascites) may also occur during recovery. A carefully planned surgical approach and close post-operative monitoring are essential to minimize these risks and support recovery.

*Q15V2. Answer at a 5^th^ grade level: What are the risks of surgery to remove my liver cancer?*

Surgery to remove liver cancer can help get rid of the cancer, but like all surgeries, it comes with some risks. One risk is bleeding, because the liver has a lot of blood vessels. Another risk is infection, which can happen after surgery and make you feel sick. Some people might have problems with how their liver works after part of it is removed, especially if the liver is already damaged. You might also feel very tired or have pain while your body heals. Doctors are very careful to keep you safe, but it's important to know these risks so you and your family can make the best decision.

*Q15V3. What could go wrong if I have surgery to take out my liver cancer?*

Having surgery to remove liver cancer can be an important and potentially life-saving treatment, but like any major operation, it carries certain risks and complications. One of the primary concerns is bleeding, since the liver has a rich blood supply and controlling bleeding during and after surgery can be challenging. There's also a risk of infection, both at the surgical site and within the abdomen. Liver function could be impacted, especially if a significant portion of the liver is removed or if the remaining liver is already damaged by disease such as cirrhosis. Other complications may include bile leakage, blood clots, or issues related to anesthesia, such as respiratory or cardiovascular problems. Recovery can take time, and some people may experience fatigue, pain, or digestive issues during the healing process. Additionally, while surgery can remove the tumor, there's always a chance that the cancer could return or spread elsewhere. It's important to discuss all these potential risks with your medical team so they can help weigh the benefits and guide you through the decision-making process.
